# Severe Acute Respiratory Syndrome Coronavirus 2 (SARS-CoV-2) in a Dog in Connecticut in February 2021

**DOI:** 10.3390/v13112141

**Published:** 2021-10-23

**Authors:** Dong-Hun Lee, Zeinab H. Helal, Junwon Kim, Amelia Hunt, Alyza Barbieri, Natalie Tocco, Salvatore Frasca, Kirklyn Kerr, Ji-Yeon Hyeon, David H. Chung, Guillermo Risatti

**Affiliations:** 1 Department of Pathobiology and Veterinary Science, University of Connecticut, Storrs, CT 06268, USA; zeinab.helal@uconn.edu (Z.H.H.); junwon.kim@uconn.edu (J.K.); amelia.hunt@uconn.edu (A.H.); natalie.tocco@uconn.edu (N.T.); frasca@uconn.edu (S.F.J.); kirklyn.kerr@uconn.edu (K.K.); jiyeon.hyeon@uconn.edu (J.-Y.H.); hyunjung.chung@uconn.edu (D.H.C.); 2 Connecticut Veterinary Medical Diagnostic Laboratory, Department of Pathobiology and Veterinary Science, University of Connecticut, Storrs, CT 06268, USA; alyza.barbieri@uconn.edu

**Keywords:** SARS-CoV-2, COVID-19, dog, sudden death, Connecticut, epidemiology, RNAscope

## Abstract

We report the first detection of severe acute respiratory syndrome coronavirus 2 (SARS-CoV-2) in a 3-month-old dog in Connecticut that died suddenly and was submitted to the state veterinary diagnostic laboratory for postmortem examination. Viral RNA was detected in multiple organs of the dog by reverse transcription real time-PCR (RT-qPCR). Negative and positive sense strands of viral RNA were visualized by in situ hybridization using RNAscope technology. Complete genome sequencing and phylogenetic analysis of the hCoV-19/USA/CT-CVMDL-Dog-1/2021 (CT_Dog/2021) virus were conducted to identify the origin and lineage of the virus. The CT_Dog/2021 virus belonged to the GH/B1.2. genetic lineage and was genetically similar to SARS-CoV-2 identified in humans in the U.S. during the winter of 2020–2021. However, it was not related to other SARS-CoV-2 variants identified from companion animals in the U.S. It contained both the D614G in spike and P323L in nsp12 substitutions, which have become the dominant mutations in the United States. The continued sporadic detections of SARS-CoV-2 in companion animals warrant public health concerns about the zoonotic potential of SARS-CoV-2 and enhance our collective understanding of the epidemiology of the virus.

## 1. Introduction

Severe acute respiratory syndrome coronavirus 2 (SARS-CoV-2) is a novel coronavirus identified as the cause of coronavirus disease 2019 (COVID-19) that caused a pandemic in late 2019 [1]. Several animal species have been reported to be susceptible to SARS-CoV-2 infection either naturally (cats, dogs, lions, minks, tigers) or after experimental infection (cats, Egyptian fruit bats, ferrets, hamsters, mice, primates, and tree shrew) [2,3,4]. In the United States, two domestic cats were confirmed to be infected with SARS-CoV-2 during April 2020, with this being the first report of SARS-CoV-2 infection in companion animals in the U.S. [5]. Since then, a total of 184 cases have been reported in cats (*n* = 87) and dogs (*n* = 97) in the U.S. as of 13 September 2021 [5].

Here, we report the first case of natural infection by SARS-CoV-2 detected in a dog in Connecticut. The virus was detected as a result of monitoring SARS-CoV-2 in dogs and cats received by the Connecticut Veterinary Medical Diagnostic Laboratory (CVMDL) for postmortem examination. On 12 February 2021, a 3-month-old female German Shepard dog was submitted to the laboratory for postmortem examination. At necropsy, the left lung was diffusely discolored purple and the heart had coalescing areas of pallor that extended into the myocardium. Eight different clinical specimens (nasal, oral, and rectal swabs, and lung, heart, kidney, spleen, and liver tissues) were collected from the dog and tested for the presence of SARS-CoV-2 RNA by reverse transcription real-time PCR (RT-qPCR) [6]. All the specimens except spleen and liver tested positive for SARS-CoV-2. Subsequently, samples were confirmed positive for SARS-CoV-2 by the USDA National Veterinary Services Laboratories. RNAscope^®^ in situ hybridization (ISH) was performed on histologic sections of lung, heart, kidney, and intestine to determine the presence of SARS-CoV-2 positive- and negative-sense RNA strands that would indicate viral presence and viral replication. Complete genome sequencing and phylogenetic analysis of the hCoV-19/USA/CT-CVMDL-Dog-1/2021 (CT_Dog/2021) virus were conducted to identify the origin and lineage of the virus.

## 2. Materials and Methods

### 2.1. RNA Extraction and Reverse Transcription Real-Time PCR (RT-qPCR)

Total RNA was extracted from nasal, oral and rectal swabs and from tissues including lung, heart, kidney, spleen, and liver using the RNeasy Plus Mini Kit (Qiagen, Valencia, CA, USA). SARS-CoV-2 RT-qPCR was performed following protocols developed by the US Centers for Disease Control and Prevention (CDC) [6].

### 2.2. Histopathology and RNAscope^®^ in Situ Hybridization

Formalin-fixed paraffin-embedded (FFPE) tissues were processed for routine hematoxylin and eosin staining for histopathological assessment.

An in-situ hybridization (ISH) assay based on RNAscope^®^ (ACD Bio-Techne, Newark, CA, USA) technology was used to visualize the presence of SARS-CoV-2 RNA in tissues [7]. The RNAscope^®^ 2.5 LS Reagent Kit-RED (cat. no. 322150) (Advanced Cell Diagnostics, Newark, CA, USA) was used with modified pretreatment conditions of FFPE tissues. Additional baking steps were added before deparaffinization, after deparaffinization, and after offline target retrieval to prevent tissue detachment. Target retrieval was performed manually using a steamer at 100 °C for 30 min. Protease III was used for 15 min at 40 °C. Each sample was quality controlled for RNA integrity with a probe specific to the housekeeping gene peptidylprolyl isomerase B (PPIB). Negative control background staining was evaluated using a probe specific to the bacterial dapB gene (Figure S1). Brightfield images were acquired using a 3DHISTECH Panoramic Scan Digital Slide Scanner using a 40× objective. Images shown were generated with SlideViewer 2.5.0.143918 software (3DHISTECH Ltd., Budapest, Hungary).

FFPE tissue (lungs, heart, kidney and intestine) cut into 4 μm sections were used to detect SARS-CoV-2. RNAscope^®^ 2.5 LS Probe-V-nCoV2019-S (antisense), 20 pairs, targeting the nCoV2019 spike gene (S) at position nt 21631 to 23303, and RNAscope^®^ 2.5 LS Probe-V-nCoV2019-orf1ab-sense, 40 pairs, targeting nsp2/nsp3 coding regions at positions 1583–4388, of SARS-CoV-2 Wuhan-Hu-1 seafood market pneumonia virus (accession number NC_045512.2) were used to detect virus negative and positive sense strands, respectively.

### 2.3. Complete Genome Sequencing and Phylogenetic Analysis

Viral RNA was amplified by multiplex RT-PCR using the QIAseq SARS-CoV-2 Primer Panel (Qiagen, Valencia, CA, USA). Next-generation sequencing (NGS) library was generated using Swift 2S™ Turbo DNA Library Kits (Swift Biosciences, Ann Arbor, MI, USA) and sequenced using the iSeq100 platform with the 300 cycle i1 Reagents v2 (Illumina). Sequencing reads were trimmed and filtered using BBDuk (https://sourceforge.net/projects/bbmap, accessed on 10 March 2021) with a minimum length 100bp and a minimum quality score of 30. Quality-filtered reads were then mapped to the reference sequence (GenBank accession no. NC045512) using minimap2 [8]. The complete genome sequence was deposited in GISAID (accession number EPI_ISL_1241386). Genetic lineage of the sample was assigned using the PANGOLIN v2.0 (https://github.com/hCoV-2019/pangolin, accessed on 10 March 2021). For phylogenetic analysis, all complete sequences of SARS-CoV-2 from companion animals in the U.S. and an additional 8–10 sequences belonging to the same genetic lineages were retrieved from the GISAID database. Maximum-likelihood (ML) phylogeny was generated using RAxML v8.2.4 and the general time-reversible nucleotide substitution model, with among-site rate variation modeled by using a gamma distributed rate heterogeneity and a proportion of invariable sites [9]. Bootstrap support values were generated using 1000 rapid bootstrap replicates. Amino acid variations were identified using the CoV-GLUE engine v1.1.107 (http://cov-glue.cvr.gla.ac.uk, accessed on 10 March 2021).

## 3. Results and Discussion

**Figure 1:**
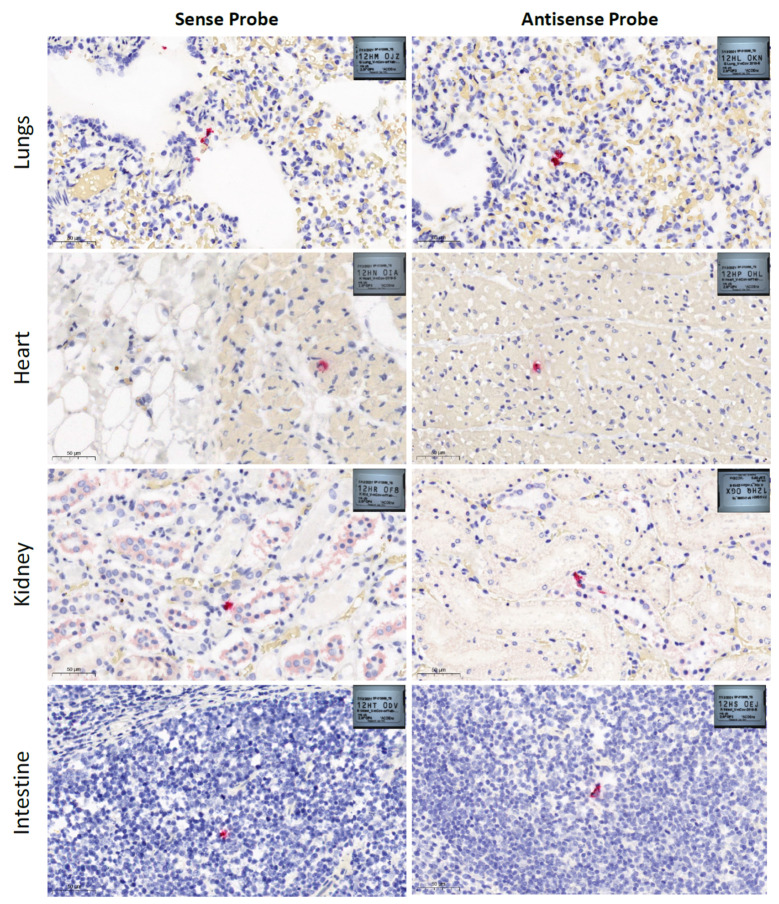
SARS-CoV-2 RNA in situ hybridization (ISH) images generated from RNAscope LS RED Singleplex ISH assays performed in formalin-fixed-paraffin-embedded (FFPE) canine tissue samples using RNAscope^®^ 2.5 LS Probe-V-nCoV2019-S and RNAscope^®^ 2.5 LS Probe-V-nCoV2019-orf1ab-sense (Advanced Cell Diagnostics, Bio-Techne, Minneapolis, MN, USA). Sections of lungs, heart, kidney, and intestine from a SARS-CoV-2 exposed dog showing red staining that represent RNAscope probes hybridized with SARS-CoV-2 positive (sense) and negative (antisense) RNA. Lungs presented marked pulmonary congestion and edema, moderate fibrin in alveoli, desquamated pneumocytes, and hyaline membranes. Heart presented epicardial edema and mild interstitial hemorrhage. No pathological changes were observed in kidneys or intestines. Hematoxylin and eosin staining, scale bars 50 µm. Images were generated with SlideViewer 2.5.0.143918 software (3DHISTECH Ltd., Budapest, Hungary).

SARS-CoV-2 RNA was detected in swabs (nasal, oral and rectal) and tissues (lung, heart, and kidney) using a RT-qPCR but was not detected in spleen and liver samples (Table 1). Low cycle threshold (Ct, 21.15 and 23.15) values were detected in nasal swabs, suggesting high virus loads in the nasal cavity most likely due to an efficient virus replication in the upper respiratory tract of the dog. Conversely, high Ct values (> 37) were detected in RNA extracted from oral and rectal swabs. The detection of SARS-CoV-2 RNA in the rectal swab, albeit at high Ct values (Table 1), suggested that virus replication may have occurred in the gastrointestinal tract of the dog, prompting us to assess virus replication in the intestine via RNAscope^®^ ISH (Figure 1).

Histopathological examination revealed marked pulmonary congestion and edema, moderate fibrin in alveoli, desquamated pneumocytes, and hyaline membranes (Figure 1). There was epicardial edema and mild interstitial hemorrhage. No pathological changes were observed in the kidney or intestine. Histologic sections of lung, heart, kidney, and intestine processed for singleplex RNAscope^®^ ISH using Probe-V-nCoV2019-S (antisense) and Probe-V-nCoV2019-orf1ab-sense demonstrated labeling indicative of viral presence and replication in a limited number of cells in all examined tissues (Figure 1). ISH data corresponded with the high RT-qPCR Ct values detected in lung, heart, kidney, and intestine (Table 1). The RT-qPCR and RNAscope^®^ ISH data suggest that the virus predominantly replicated in the upper respiratory tract, but viral replication was absent or very low in other organs.

A total of 2,120,432 NGS reads were assembled into a single consensus sequence with 100% coverage of the reference and high mean depth of coverage (10,054.9). The genome sequence of CT-dog/2021 virus was assigned as B1.2. by PANGOLIN and GH by GISAID classification. BLAST search results in the GISAID database indicated that the virus shared > 99.97% nucleotide identity with SARS-CoV-2 identified in the U.S. during the winter of 2020–2021 (Table 2).

**Figure 2:**
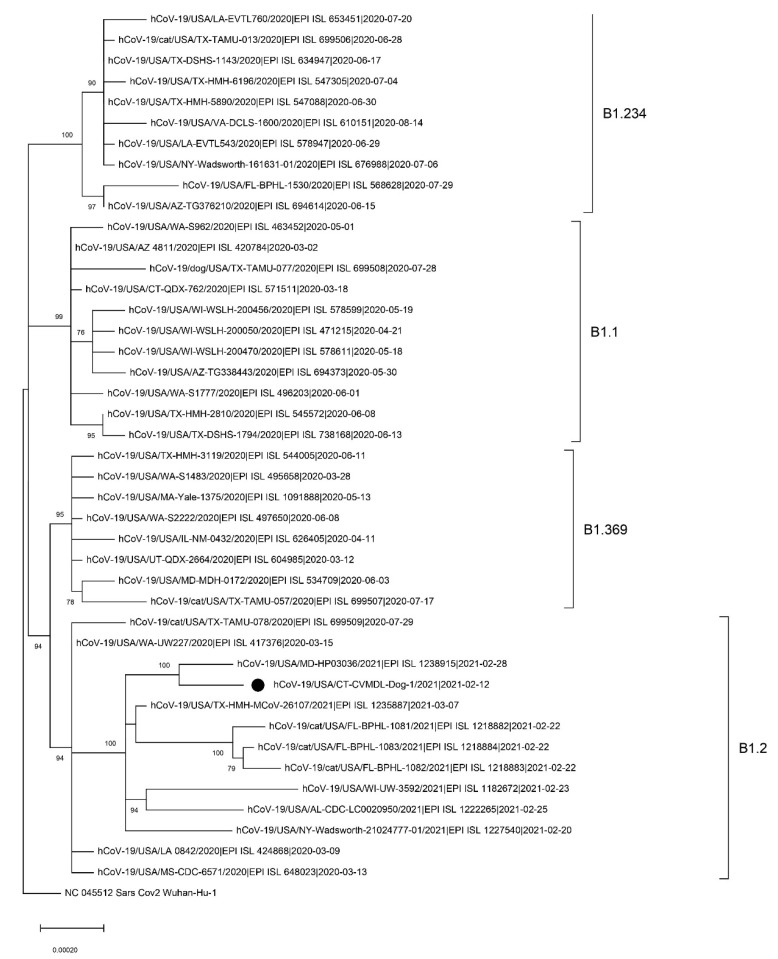
Maximum-likelihood phylogenetic tree of the full-length SARS-CoV-2 genome sequences. The tree was rooted to the Wuhan-Hu-1 virus. Bootstrap values over 70% are shown next to the branches. Scale bar indicates nucleotide substitutions per site. The black circle identifies the hCoV-19/USA/CT-CVMDL-Dog-1/2021 (CT_Dog/2021) virus. The genetic lineages assigned using the PANGOLIN v2.0 (https://github.com/hCoV-2019/pangolin, accessed on 10 March 2021) are indicated with brackets.

In the ML phylogeny, the CT_Dog/2021 virus belonged to the B1.2. lineage. It was not clustered with other SARS-CoV-2 identified from companion animals in the U.S. (Figure 2).

We found amino acid substitutions in Spike (D614G), N (D377Y, P67S, P199L), NS3 (G172V, Q57H), NS8 (S24L) NSP2 (T85I), NSP4 (M458I), NSP5 (L89F), NSP12 (P323L), NSP14 (N129D), and NSP16 (R216C) proteins. The virus from this CT dog did not contain mutations related to the South African variant B.1.351 (N501Y, E484K and K417N in Spike) or the U.K. variant B.1.1.7 (69/70 deletion, N501Y, and P681H in Spike). The B.1 and its sub-lineages that carry both D614G in spike and P323L in nsp12 substitutions have become the dominant variants across the world [10]. The D614G and P323L occurred in China on 24 January 2020 and in the U.K. on 3 February 2020, respectively. Both mutations were first detected in the U.S. on 28 February 2020 and have since become the dominant mutations in the U.S. [11].

The role of companion animals in the evolution and spread of SARS-CoV-2 remains uncertain. Although we did not find direct evidence for transmission of the virus between the owner and the dog in this case, it has been reported that SARS-CoV-2 has repeatedly spilled over from humans to companion animals, highlighting the need for enhanced surveillance in animals. It is concerning that companion animals could become reservoir species of SARS-CoV-2 since they are susceptible to infection and could excrete infectious virus [12].

## Figures and Tables

**Table 1:** Detection of SARS-CoV-2 by RT-qPCR and ISH RNAscope in swabs and tissue samples collected from a dog.

Sample/Tissue	SARS-CoV-2 RT-qPCR (Ct Value)		ISH RNAscope	
	N1 Probe	N2 Probe	Sense Probe Signal	Antisense Probe Signal
Nasal swab	21.15	23.15	-	-
Oral swab	37.5	43.08	-	-
Rectal swab	39.1	38	-	-
Lung tissue	38.7	39	Detected	Detected
Heart tissue	36.57	38.36	Detected	Detected
Kidney tissue	38.76	39.16	Detected	Detected
Spleen tissue	not detected	not detected	-	-
Liver tissue	not detected	not detected	-	-
Intestine tissue	-	-	Detected	Detected

**Table 2:** SARS-CoV-2 sharing the highest nucleotide identity found by BLAST search in GISAID database on 14 March 2021.

GISAID Accession	Virus	Location	Collection Date (YYYY-MM-DD)	Sequence Identity
EPI_ISL_1137193	hCoV-19/USA/PA-MGEL-01496/2021	USA/Pennsylvania	2021-01-25	99.99%
EPI_ISL_853340	hCoV-19/USA/PA-MGEL-01148/2020	USA/Pennsylvania	2020-12-02	99.99%
EPI_ISL_1137240	hCoV-19/USA/PA-MGEL-00902/2020	USA/Pennsylvania	2020-10-30	99.98%
EPI_ISL_1202236	hCoV-19/USA/TX-HMH-MCoV-16526/2020	USA/Texas	2020-11-03	99.98%
EPI_ISL_1094242	hCoV-19/USA/FL-CDC-2-3847117/2021	USA/Florida	2021-01-20	99.98%
EPI_ISL_1080077	hCoV-19/USA/TX-HMH-MCoV-25439/2021	USA/Texas	2021-01-21	99.98%
EPI_ISL_1075269	hCoV-19/USA/TX-HMH-MCoV-20826/2021	USA/Texas	2021-01-02	99.98%
EPI_ISL_1049241	hCoV-19/USA/UT-UPHL-2102150865/2021	USA/Utah	2021-02-02	99.97%
EPI_ISL_783726	hCoV-19/USA/TX-HMH-MCoV-19267/2020	USA/Texas	2020-11-24	99.97%
EPI_ISL_1095270	hCoV-19/USA/AZ-CDC-2-3846423/2021	USA/Arizona	2021-01-14	99.97%

## Data Availability

The complete genome sequence has been deposited in GISAID (accession number EPI_ISL_1241386).

## Supplementary Materials

The following are available online at www.mdpi.com/article/10.3390/v13112141/s1, Figure S1: In situ hybridization (ISH) images generated from RNAscopeLS RED Singleplex ISH Assays in formalin-fixed-paraffin-embedded (FFPE) canine tissue samples.
