# Supplementary material for: Severe Acute Respiratory Syndrome Coronavirus 2 (SARS-CoV-2) in a Dog in Connecticut in February 2021
Source: Viruses. 2021 Oct 23;13(11):2141. doi: 10.3390/v13112141 (PMC8623824; doi:10.3390/v13112141)
Supplement: Supplementary file 1 [file viruses-13-02141-s001.zip › viruses-1166844-supplementary.pdf]

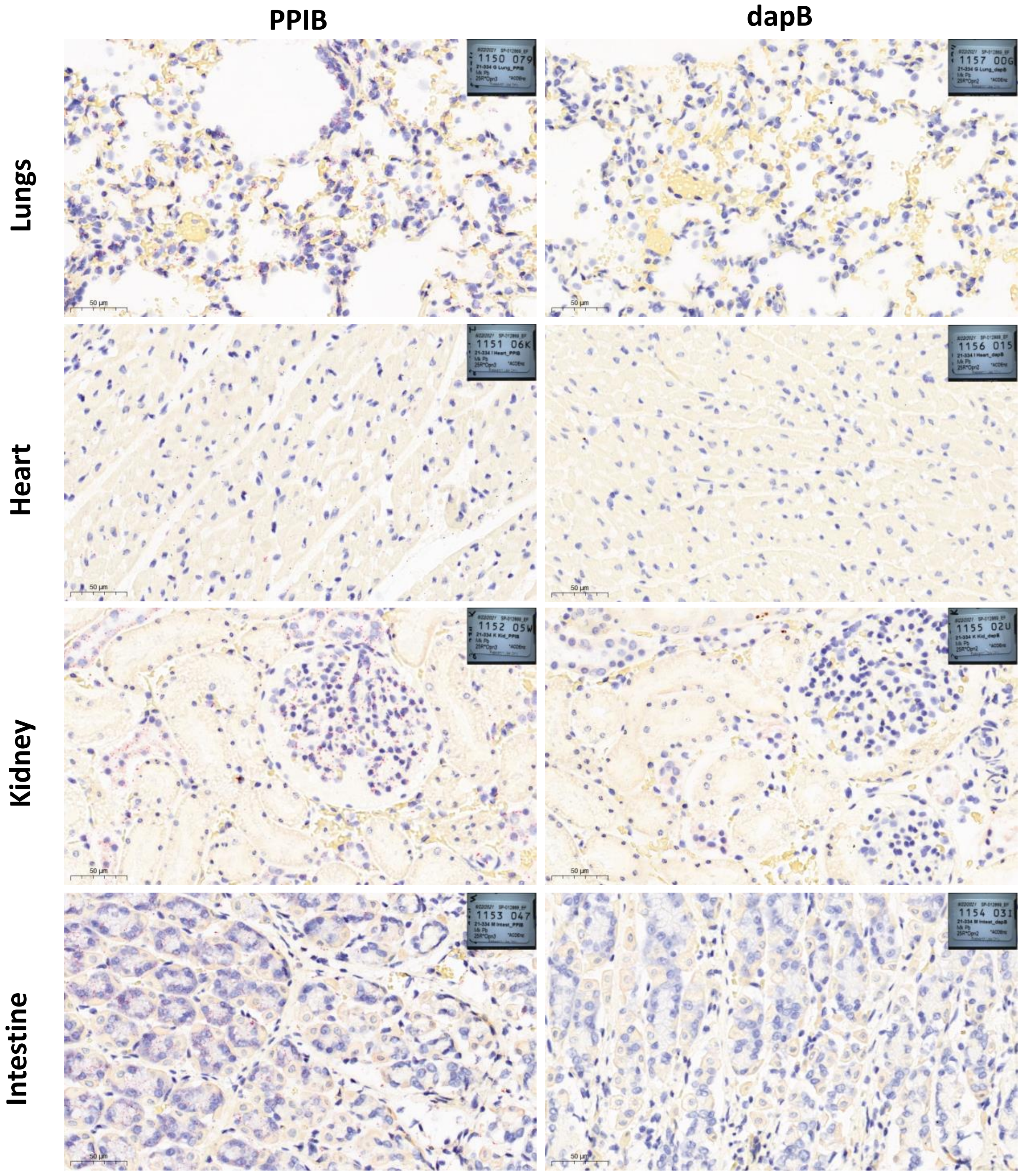

Figure S1: In situ hybridization (ISH) images generated from RNAscopeLS RED Singleplex ISH Assays in formalin-fixed-paraffin-embedded (FFPE) canine tissue samples. Evaluation of tissue samples and quality of RNA in FFPE tissues (lungs, heart, kidney and intestine) collected from a dog naturally exposed to SARS-CoV-2, using positive and negative reference controls. ACD positive control probe CI-PPIB detects *Canis lupus familiaris* peptidylprolyl isomerase B (cyclophilin B) mRNA (ACD cat # 437448) and ACD negative control probe dapB *Bacillus subtilis* gene dihydrodipicolinate reductase mRNA (ACD cat # 312038). Hematoxylin and eosin staining, scale bars 50 μm. Images were generated with SlideViewer 2.5.0.143918 software (3DHISTECH Ltd., Budapest, Hungary).
